# Integrated physiological and transcriptomic analyses reveal the molecular mechanism behind the response to cultivation in *Quercus mongolica*

**DOI:** 10.3389/fpls.2022.947696

**Published:** 2022-08-08

**Authors:** Min Jiang, Xinman Li, Yangchen Yuan, Guowei Zhang, Jiushuai Pang, Junjie Ren, Jinmao Wang, Minsheng Yang

**Affiliations:** ^1^College of Forestry, Hebei Agricultural University, Baoding, China; ^2^Hongyashan State-Owned Forest Farm, Baoding, China

**Keywords:** *Quercus mongolica*, cultivation measures, physiology, transcriptome analysis, WGCNA

## Abstract

*Quercus mongolica*, a common tree species for building and landscaping in northern China, has great commercial and ecological value. The seedlings of *Q. mongolica* grow poorly and develop chlorosis when introduced from high-altitude mountains to low-altitude plains. Effective cultivation measures are key to improving the quality of seedlings. To investigate the complex responses of *Q. mongolica* to different cultivation measures, we compared the adaptability of 3-year-old *Q. mongolica* seedlings to pruning (P), irrigation (W), and fertilization [F (nitro compound fertilizer with 16N-16P-16K)]. Physiological measurements and transcriptome sequencing were performed on leaves collected under the P treatments (control, cutting, removal of all lateral branches, and removal of base branches to one-third of seedling height), the W treatments (0, 1, 2, 3, 4, or 5 times in sequence), and the F treatments (0, 2, 4, and 6 g/plant). Analyses of the physiological data showed that P was more effective than W or F for activating intracellular antioxidant systems. By contrast, W and F were more beneficial than P for inducing the accumulation of soluble sugar. OPLS-DA identified superoxide dismutase, malondialdehyde, and peroxidase as critical physiological indices for the three cultivation measures. Transcriptome analyses revealed 1,012 differentially expressed genes (DEGs) in the P treatment, 1,035 DEGs in the W treatment, and 1,175 DEGs in the F treatment; these DEGs were mainly enriched in Gene Ontology terms related to the stress response and signal transduction. Weighted gene coexpression network analyses indicated that specific gene modules were significantly correlated with MDA (one module) and soluble sugar (four modules). Functional annotation of the hub genes differentially expressed in MDA and soluble sugar-related modules revealed that *Q. mongolica* responded and adapted to different cultivation measures by altering signal transduction, hormone levels, reactive oxygen species, metabolism, and transcription factors. The hub genes HOP3, CIPK11, WRKY22, and BHLH35 in the coexpression networks may played a central role in responses to the cultivation practices. These results reveal the mechanism behind the response of *Q. mongolica* to different cultivation measures at the physiological and molecular levels and provide insight into the response of plants to cultivation measures.

## Introduction

Quercus species are the most diverse and widely distributed species in Fagaceae, which are dominant in the temperate deciduous broadleaved forests of China (Chen, 2020). *Quercus mongolica* is the main industrial timber forest species in China and is an essential eco-commercial tree species. Its ecological value is reflected in good corrosion resistance and soil and water conservation with high development and utilization value. *Quercus mongolica*, the most cold-resistant and drought-tolerant tree species in the deciduous oak family (Chen et al., 2018a), is mainly distributed in northeastern China, including parts of Heilongjiang, Jilin, Liaoning, and eastern Inner Mongolia. This species grows at a vertical elevation of 300–1,300 m in the mountains. The *Q. mongolica* National Forest Germplasm Resource Bank is located in Yi County, northwest Baoding, Hebei Province, where it belongs to a low elevation plain landform (Zhao, 2006). By contrast, many Mongolian oak habitats are colder and drier (Sun et al., 2019). Some Mongolian oak (*Q. mongolica*) germplasm resources collected and preserved at the Germplasm Resource Bank are poorly adapted with poor growth and chlorosis at the seedling stage. Therefore, it is essential to explore effective cultivation measures for *Q. mongolica* in low-elevation plain areas to increase its popularity and application in northern China.

The quality of *Q. mongolica* is not only determined by its genetic characteristics but also closely related to cultivation and management measures. Pruning is a common and valuable technique for rejuvenating plants (Zhang et al., 2020b). When selected stems and branches are removed, trees can be cultivated for their structural shape and branching (Alejandra Giantomasi et al., 2015), reduce shoot competition, and redistribute assimilates (increase the photosynthetic capacity of remaining leaves) to increase yield (Heichel and Turner, 1983). The accumulation of organic matter in plants originates from photosynthesis, and a change in soil water content directly affects the photosynthetic capacity of plant leaves (Dong and Li, 2013). When the water content is below or above the normal demand range of the plant, it will experience water stress. Plants protect themselves from stress by changing stomatal regulation of leaves, and the activities of antioxidant enzymes change accordingly (Wang et al., 2013; Kang, 2020). Therefore, improving the irrigation strategy improves the quality and yield of seedlings and can minimize water consumption. Nutrients are the material basis of forest growth. Fertilization is an effective method of promoting the growth and storage of nutrients in young trees and improving the survival rate of afforested areas. Studies on *Quercus variabilis* and *Q. mongolica* have shown that fertilization improves nutrient storage and balance in tissues and roots and contributes to stress resistance (Andivia et al., 2012; Sun et al., 2021). However, there was a threshold for the promotion of seedling growth by soil fertility. Uscola’s study showed that the *Quercus ilex* L. seedling biomass was the highest when the nitrogen application rate was 125 mg/plant, and the seedling was poisoned when the nitrogen application rate was over 200 mg/plant (Uscola et al., 2014).

It is expected that *Q. mongolica* will show various responses to different cultivation measures. If a plant cannot acclimate to the cultivation treatments that exceed its normal growth level, it will experience stress; therefore, plants employ many acclimatization mechanisms to ensure survival (Guo et al., 2021). For instance, they will activate a series of defense mechanisms regulated by antioxidant enzyme systems, osmotic regulators, and structural substances in the membrane (Zhu, 2016). A range of downstream physiological and metabolic products are activated by many stress-responsive genes to synthesize a variety of functional proteins through a complex signal transduction network to confer tolerance to environmental stress (You and Chan, 2015). Ca^2+^ is a major secondary messenger in plant signal transduction mediated stress and development (Liese and Romeis, 2013). The stimuli triggered by external adverse factors can generate diverse Ca^2+^ changes, and these changes can be recognized and sensed by specific calcium sensors/receptors to induce further transcriptional and metabolic responses (Shao et al., 2008; Shi et al., 2018). Numerous studies have shown that other second messengers such as ROS (reactive oxygen species) are also play an important role in stress signal transduction (Shukla et al., 2017; Czarnocka and Karpinski, 2018; Krasensky-Wrzaczek and Kangasjarvi, 2018; Sasidharan et al., 2018). However, these molecules also react directly with DNA, proteins, and lipids, causing severe damage to cells. Therefore, aerobic organisms strictly control ROS concentrations through a ROS elimination pathway composed of enzyme and non-enzyme antioxidants (Gill and Tuteja, 2010). Although the integrated regulation induced by *Q. mongolica* in different measures has important breeding references, the molecular basis of its effect is poorly understood. Here we report physiological and transcriptome responses of *Q. mongolica* to different cultivation measures, including pruning (P), irrigation (W), and fertilization [F (nitro compound fertilizer with 16N-16P-16K)]. Quantitative changes in physiological substances, such as soluble sugars, soluble proteins, superoxide dismutase (SOD), peroxidase (POD), catalase (CAT), and malondialdehyde (MDA), were compared by the chemometrics method to screen out important metabolic regulators. Gene expression profiles were created for the different treatments to identify potentially important genes in the response of *Q. mongolica* to different cultivation practices. In addition, weighted gene coexpression network analysis has been extensively used in the analysis of correlation between traits and modules in plant research (Liu et al., 2019), coexpression networks among important genes were established based on WGCNA, and the hub genes that may play a central role in the induction of three cultivation measures in *Q. mongolica* were isolated. The results provide a basis for regulating the response of three cultivation measures in *Q. mongolica* and provide insight into the molecular mechanism behind the response of *Q. mongolica* to cultivation measures.

## Materials and methods

### Plant material and treatments

The experiments were conducted at the Qiliting demonstration farm (39°21′35′N, 115°33′49′E) at the Hongyashan State-Owned Forest Farm Management Bureau in Hebei Province. The site has a warm, continental monsoon climate, with an average annual air temperature of 11.9°C. Average temperatures in January and July are −3°C and 27.5°C, respectively. The annual mean humidity, annual precipitation and annual evaporation are 40%, 553.9 mm and 1162.70 mm, respectively. Frost-free periods typically last from 170 to 185 days and total solar radiation ranges from 116 to 136 C/cm^2^. Provenance seeds of *Q. mongolica* were collected from Xiaoqinling Mountain in Lingbao City, Sanmenxia City, Henan Province. The 3-year-old seedlings were planted in the same nursery and the developmental stage of the seedlings at the beginning of the experiment are shown in Supplementary Figure S1. The P test was conducted at the beginning of March 2021 with four treatments: control [no pruning (Pck)], cutting (P1), removal of all lateral branches (P2), and removal of the base to one third of the lateral branches (P3). The W experiment was performed in early April 2021. Six plots with the same conditions were selected. Each plot had an area of 75 m^2^ (15 × 5 m), and five W events were performed from April to August. The amount of water supplied was up to 95% of the designed experimental field capacity. One fewer experimental field was irrigated each month. There were six treatments: control [no irrigation (Wck)], irrigate once (W1), irrigate twice (W2), irrigate three times (W3), irrigate four times (W4), and irrigate five times (W5). The fertilizer (F) experiment was performed at the end of May 2021. Nitro compound fertilizer (16N-16P-16K) was used for the four treatments: control [no fertilizer (Fck)], 2 g/plant (FA), 4 g/plant (FB), and 6 g/plant (FC).

All experiments were performed in a random block design, and 10 seedlings of relatively uniform growth with no diseases or insect pests were selected for each of the three repeated blocks.

### Growth indices

Five seedlings of uniform growth were selected for each treatment, and the average value was obtained after three repetitions. Plant height was measured with a tape measure, and the diameter at the ground was measured with a Vernier scale. At the end of the experiment, the fourth or fifth leaf of the main branch was collected from top to bottom in the same position with three replicates. The leaves were cleaned and dried, and fresh weight was measured. The leaves were imaged with a Canoscan LIDE300 scanner and saved as electronic images. The leaves were placed in an oven at 105°C for 15 min and dried at 80°C to a constant weight. After the leaves cooled, their dry weight was measured. Lamina software was used to determine the leaf area.

### Physiological indices

Soluble protein content was determined by Coomassie Brilliant Blue G-250 staining (Bradford, 1976). Briefly, the leaf was frozen in liquid nitrogen and pulverized, then 200 mg fine powder was extracted in 6 ml distilled water. The samples were rotated for 10 min at 4°C. The absorbance of a mixture of the protein extract and Coomassie Brilliant Blue reagent was determined at 595 nm with a spectrophotometer and pure reagent as the blank control.

Soluble sugar content was determined by anthrone colorimetry (Jan and Roel, 1993). The leaf was frozen in liquid nitrogen and pulverized, then 200 mg fine powder was extracted in 4 ml of 80% ethanol in an ultrasonic bath for 30 min at 80°C. The solution was centrifuged for 10 min at 10,000 rpm. The pellet was extracted twice and the supernatants were combined, then 5 ml anthrone sulfate was added and the solution was heated in boiling water for 10 min, and cooled. Absorbance was measured at 620 nm with a spectrophotometer.

Malondialdehyde content was determined using the thiobarbituric acid (TBA) method as described by Shukla et al. with minor modifications (Shukla et al., 2012). The leaf was frozen in liquid nitrogen and pulverized, then 200 mg fine powder was extracted in 5 ml of 5% (w/v) TCA. The samples were rotated for 10 min at 4°C. A mixture of the MDA extract and 0.6% TBA was heated in boiling water for 15 min and centrifuged at 12,000 r/min for 10 min at 4°C. The absorbance of the supernatant was measured at 532, 600, and 450 nm.

Superoxide dismutase, POD, and CAT activity was determined by the method described by Xu et al. (2020) with minor modifications. For each sample, 200 mg fresh tissue was ground in a pre-chilled mortar and homogenized in 5 ml phosphate buffer (50 mmol/L, pH 7.8). The solution was centrifuged at 4°C for 20 min at 12,000 rpm, and then take the supernatant as the extract of enzyme. SOD activity was measured at 560 nm in a 3 ml reaction mixture containing 0.3 ml 130 mmol/L methionine, 0.3 ml 750 μmol/L NBT, 0.3 ml 1 μmol/L EDTA-Na_2_, 0.3 ml 0.2 μmol/L riboflavin, 1.8 ml 50 mmol/L phosphate buffer, and the extract. After dissolving 0.11 ml enzyme extract in 2.89 ml mixture (containing 2.5 ml guaiacol, 0.17 ml 250 mmol/L H_2_O_2_, and 0.22 ml distilled water), we calculated POD activity by measuring absorbance at 470 nm. After adding 200 μl enzyme extract to 2.8 ml 20 mmol/L H_2_O_2_ mixture, we measured absorbance at 240 nm to calculate CAT activity.

The samples were immediately frozen in liquid nitrogen and stored at −80°C for later determination of physiological indicators and RNA-seq assay. Five seedlings of relatively uniform growth were selected. The fourth or fifth mature leaf on the main branch from top to bottom in the same position was taken together as a biological repeat, there were 15 seedlings with three biological replicates for each treatment. We measured all indices by mixing and weighing 200 mg leaf blades.

### RNA extraction, transcriptome sequencing, and data analysis

Samples collected at the same time and from the same batch were used for transcriptome sequencing. A total of 33 independent RNA-seq libraries were constructed and sequenced from the Pck, P1, P2, P3, Wck, W2, W5, Fck, FA, FB, and FC treatments. Three representative samples, such as Wck, W2, and W5, were selected for further transcriptome analysis according to phenotypic and physiological changes. Each group consisted of three biological replicates. Total RNA was extracted from each sample according to the instructions for the Trizol reagent kit (Invitrogen, Carlsbad, CA, United States). RNA quality was evaluated with an Agilent 2100 Bioanalyzer (Agilent Technologies, Palo Alto, CA, United States) and checked with RNase-free agarose gel electrophoresis. Then 33 cDNA libraries were prepared and transcriptome sequencing was completed by Gene Denovo Biotechnology (Guangzhou, China) using the Illumina Hiseq4000 platform. The RNA-Seq raw sequencing datasets were submitted to the NCBI Biological Project Database with an SRA accession number SUB11496576. The raw reads from transcriptome sequencing were filtered with Fastp (v0.18.0) to obtain high-quality clean reads (Chen et al., 2018b), and the mapped reads from each sample were assembled with StringTie v1.3.1 (Pertea et al., 2016). The FPKM value was calculated with StringTie (max_memory, 30G; seqType, fq; CPU, 10; KMER_SIZE, 31; min_kmer_cov, 9; normalize_reads; normalize_max_read_cov, 50) and used to identify differentially expressed genes (DEGs). To assess the metabolic pathways and related gene functions, we performed Gene Ontology (GO) and Kyoto Encyclopedia of Genes and Genomes (KEGG) enrichment analyses using the DEGs (*q* ≤ 0.05).

### WGCNA

WGCNA was performed with OmicShare tools^1^ to identify the key regulatory genes in *Q. mongolica* that responded to the three cultivation practices. An adjacency matrix of differential genes was constructed with a threshold power of 6, and a dynamic tree cut procedure (minimum module size = 50, merge cut height = 0.1) was used to screen similar modules in the hierarchical tree. The modular eigengene was defined as the first principal component of a given module and was used to represent the expression profile of the module gene in each sample. Pearson correlation analyses of gene significance (GS; representing the correlation between each gene and the trait of interest) and module membership (MM; representing the correlation of the expression of each gene to the corresponding module eigengene) were performed. The higher the correlation, the greater the role of the gene’s biological function in the corresponding module. Genes with MM > 0.8 and GS > 0.6 were selected as the hub genes of the module, which represented the expression trend for the entire module. We constructed and visualized the coexpression networks using Cytoscape (v3.7.1; Shannon et al., 2003).

### Quantitative real-time polymerase chain reaction analysis of hub genes

Quantitative real-time polymerase chain reaction was performed to validate the expression of genes from the coexpression network. An M5 sprint qPCR RT kit with gDNA remover (Mei5 Biotechnology, Beijing, China) was used to synthesize the cDNA. qRT-PCR was performed with MagicSYBR Mixture (CWBIO, Beijing, China) on a StepOnePlus Real-Time PCR System (Thermo Fisher Scientific, Wilmington, DE, United States). The β-actin gene was used as the internal control (Chatwin et al., 2014). The primers were designed in Primer Premier 6.0 (Palo Alto, CA, United States). Relative expression was calculated with the 2^−ΔΔCT^ method (Livak and Schmittgen, 2001).

### Statistical analysis

Statistical analyses of the leaf physiological data were performed with SPSS Statistics23 (IBM, Armonk, NY, United States). Analyses of variance and Duncan’s test were used to detect differences between samples (*p* < 0.05). Data standardization and principal component analyses (PCA) were performed in Xu et al. (2015) (Originlab, Northampton, MA, United States). Orthogonal partial least squares discriminant analyses (OPLS-DA) were performed using online software.^2^ A cluster heat map was prepared with TBtools (Chen et al., 2020), and the KEGG enrichment loop map was prepared with the Sanger Box 3.0 online tool.

## Results

### Morphological and physiological characteristics affected by cultivation measures

**Figure 1:**
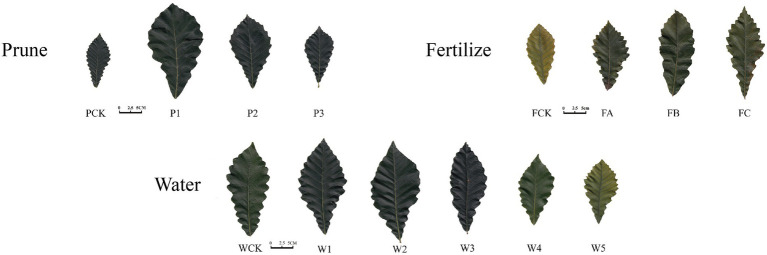
Morphological change in *Quercus mongolica* leaves under different cultivation measures. Prune is pruning, Fertilize is fertilization, and Water is irrigation.

**Figure 2:**
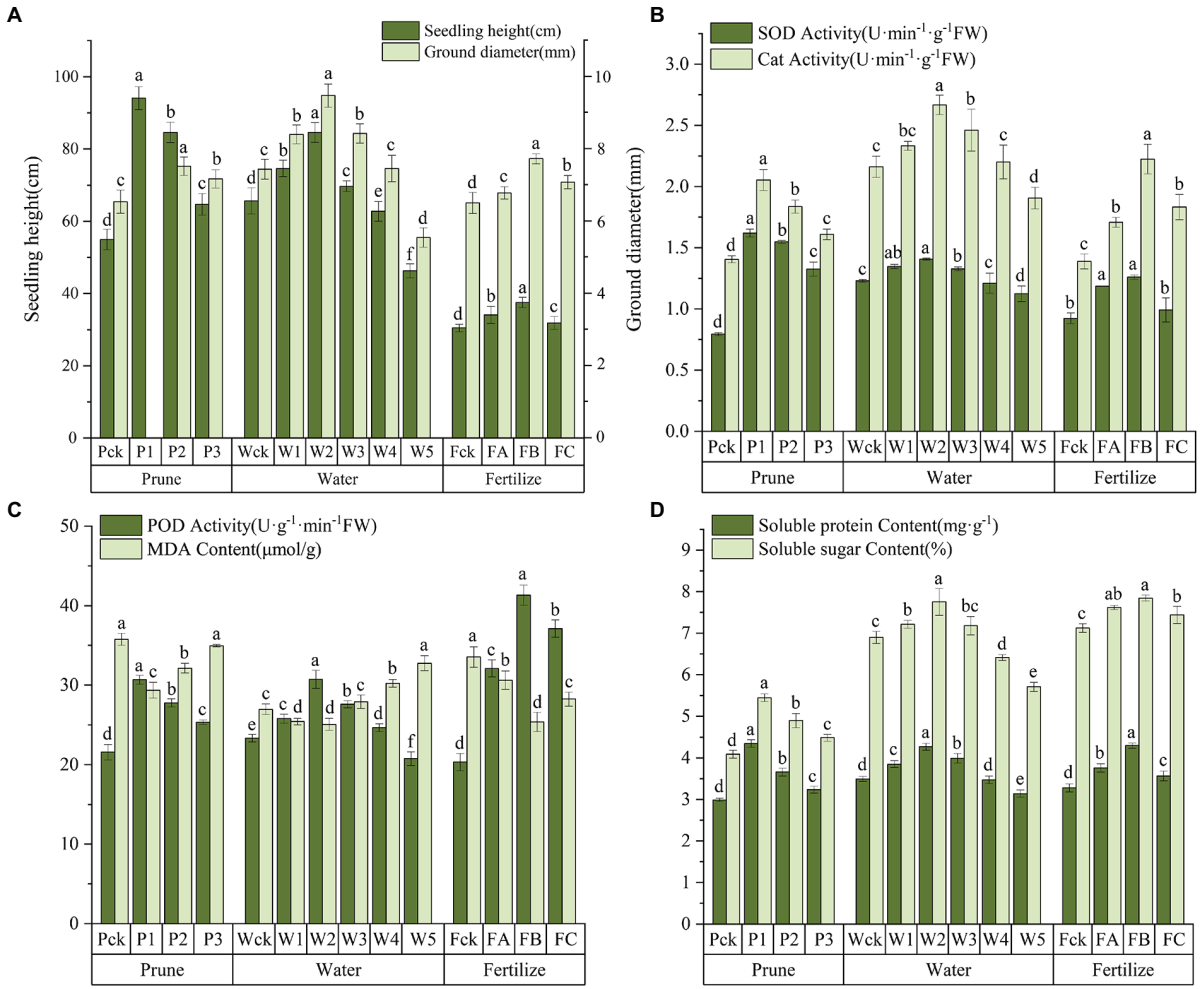
**(A)** Growth in plant height and ground diameter. **(B)** SOD and CAT activity. **(C)** POD and MDA content. **(D)** Soluble protein and soluble sugar content. Error bars indicate the standard error, and different lowercase letters indicate significant differences between treatments (*p* < 0.05).

The leaf morphology of *Q. mongolica* grown under the different cultivation measures varied (Figure 1). The P treatment had little effect on leaf color, but significant differences were detected in leaf area and leaf biomass. The leaves were different as the W amount increased. Water loss and discoloration appeared at W4. The leaf color of the plants grown in the F treatment was dark green under FB and yellow-green under the other treatments. The leaf area and leaf biomass of the P1, W2, and FB treatments were significantly greater than those of the other treatments (Table 1). The increases in height and diameter of the *Q. mongolica* seedlings before and after the cultivation measures were analyzed and compared (Figure 2A). The height and diameter of *Q. mongolica* seedlings under the W and F treatments first increased and then decreased. The fastest growth rate occurred in P1. The average growth was 94.1 cm, and the growth in seedling height was smallest in the F treatment. The largest increase in ground diameter occurred in W2 (P1 stubble was buried after sprouting, so ground diameter was not recorded), and the average increase was 9.48 mm.

**Table 1:** Morphological change in leaves under different treatments.

Cultivation measure	Treatment	Leaf area/cm^2^	Leaf dry matter/g
Prune	Pck	38.340 ± 2.767d	0.355 ± 0.026d
	P1	136.081 ± 3.394a	1.374 ± 0.057a
	P2	65.364 ± 2.757b	0.718 ± 0.027b
	P3	44.053 ± 2.293c	0.427 ± 0.018c
Water	Wck	72.329 ± 3.389e	0.763 ± 0.041c
	W1	83.680 ± 3.208b	0.833 ± 0.042b
	W2	91.576 ± 1.914a	0.940 ± 0.023a
	W3	79.108 ± 2.709c	0.825 ± 0.016b
	W4	74.219 ± 2.218d	0.763 ± 0.024c
	W5	69.865 ± 3.314f	0.680 ± 0.021d
Fertilize	Fck	54.268 ± 2.138d	0.581 ± 0.024c
	FA	56.707 ± 2.572c	0.591 ± 0.031c
	FB	67.449 ± 1.053a	0.762 ± 0.042a
	FC	61.959 ± 2.734b	0.689 ± 0.029b

Data are means ± standard deviation. Different lowercase letters indicate significant differences between treatments (*p* < 0.05).

To explore the cellular responses of *Q. mongolica* to oxidative and osmotic damage, we determined POD, SOD, and CAT activity as well as MDA, soluble protein, and soluble sugar content in leaves (Figures 2B–D; Supplementary Table S1). SOD, POD, and CAT activity increased in the P treatment with increases in P intensity and reached the maximum value at P1. SOD activity was higher in P1 than in the W and F samples. SOD, POD, and CAT activity first increased in the W and F treatments and then decreased, reaching the maximum value in W2 and FB. SOD, POD, and CAT activity was significantly lower in W5 than in the control. The *Q. mongolica* seedlings treated with W experienced more extensive damage to the cell membranes and had a greater increase in MDA concentration. W3, W4, and W5 increased by 3.49%, 12.17%, and 21.48%, respectively, compared to Wck. MDA content in P and F was lower than in ck. Soluble protein and soluble sugar content first increased and then decreased, reaching the maximum value in P1, W2, and FB. Note that the soluble sugar content was much higher in the W and F samples than in the P samples.

**Figure 3:**
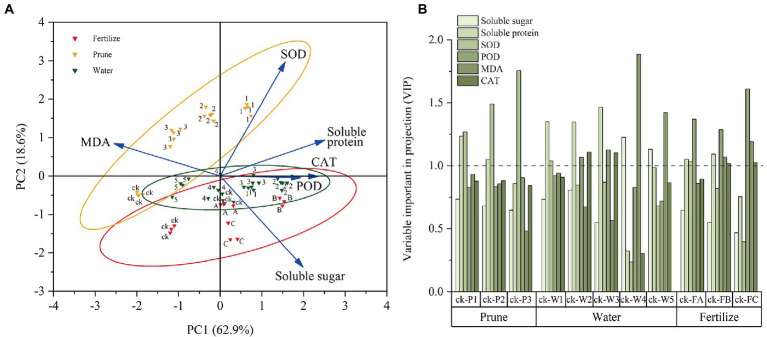
Chemometrics analysis of the physiological data. **(A)** Principal component analysis (PCA) of the physiological indices. **(B)** Orthogonal partial least squares discriminant analysis (OPLS-DA) for the physiological parameters.

The physiological test data were standardized for the chemometrics analyses, and the samples and physiological indices were grouped by PCA (Figure 3A). The total PC1 variance was 62.9%, indicating sample separation. The P-treated samples were divided into three groups: Pck, P1, and P2–P3. The W-treated samples were divided into three groups: Wck-W1 and W3–W4, W2, and W5. The F-treated samples were divided into Fck, FA-FC, and FB. All P-treated samples except Pck were on the positive axis of PC2 (18.6%). Most W-and F-treated samples were on the negative axis, but some samples, such as W3 and W4, were on the positive axis. PC1 differentiated the samples from the different treatments and cultivation measures, and PC2 determined the discrepancies between the samples caused by the different cultivation measures. In addition, PC1 had positive loadings from soluble sugar, soluble protein, SOD, CAT, and POD but a negative loading from MDA. PC2 had positive loadings from SOD, MDA, and soluble protein and negative loadings from CAT, POD, and soluble sugar.

The importance of the *Q. mongolica* physiological indices under the three cultivation measures was evaluated by OPLS-DA. The variable importance in projection threshold (VIP = 1) was used as the intensity measurement for the physiological indices (Figure 3B). Soluble protein (P1-P2) and SOD were important physiological indices in the P test. Soluble sugar (W4-W5), soluble protein (W1-W2), SOD (in W1), POD (W2-W3), MDA (W4-W5), and CAT (W2-W3) were important physiological indices for the W test. Soluble protein (FA-FB), SOD (in FA), POD, MDA (FB-FC), and CAT (FB-FC) were important physiological indices for the F test.

### *Quercus mongolica* gene expression profiles under different cultivation measures

**Figure 4:**
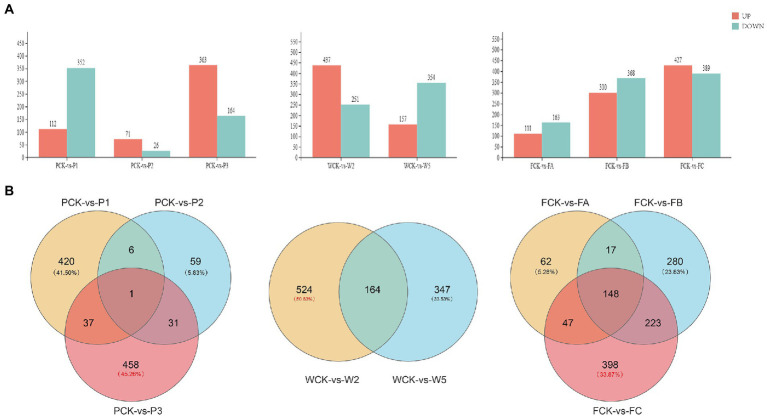
Differentially expressed genes (DEGs) in the leaves of *Quercus mongolica* under different cultivation measures. **(A)** The number of DEGs. **(B)** A Venn diagram representation of the number of DEGs.

The response of *Q. mongolica* DEGs to the three treatments was analyzed in DEseq2 software with a false discovery rate (FDR) threshold <0.05 and an absolute fold change ≥1.5. The distribution of DEGs is shown in Figure 4A. Among the different P methods, 464 DEGs (112 upregulated and 352 downregulated), 97 DEGs (71 upregulated and 26 downregulated), and 527 DEGs (363 upregulated and 164 downregulated) were detected in the P1, P2, and P3 groups, respectively. Among the different W treatments, 688 DEGs (437 upregulated and 251 downregulated) and 511 DEGs (157 upregulated and 354 downregulated) were detected in the W2 and W5 groups, respectively. A total of 274 DEGs (111 upregulated and 163 downregulated), 668 DEGs (300 upregulated and 368 downregulated), and 816 DEGs (427 upregulated and 389 downregulated) were detected in FA, FB, and FC. A Venn diagram (Figure 4B) analysis showed that 458 DEGs were specifically expressed in the PCK-P3 group, and 524 DEGs were specifically expressed in the WCK-W2 group. A total of 398 DEGs were expressed in the FCK-FC group, accounting for 45.26% (1,012), 50.63% (1,035), and 33.87% (1,175) of the total DEGs, respectively. According to the differences in the number of DEGs, different W amounts seemed to be a greater driving force behind the change in transcriptome level. In addition, the detected DEGs had different expression patterns in different sample groups. These findings suggest that *Q. mongolica* responded to the stress of the different cultivation measures with complex transcriptional regulation.

Gene Ontology enrichment analyses were performed to understand the biological functions of the DEGs expressed by the different cultivation measures. The DEGs were divided into three functional categories: biological processes (BP), cell components (CC), and molecular functions (MF). The prominent GO terms in all comparable groups were “metabolic processes” in BP, “cells” in CC, and “catalytic activity” in MF. Many DEGs were involved in processes related to the stress response, such as “response to a stimulus” (185, 15, 168, 273, 160, 114, 236, and 259 genes, respectively) and “immune system processes” (23, 3, 26, 49, 13, 15, 24, and 29 genes, respectively; Supplementary Figure S1). The results of KEGG functional annotation indicated that many DEGs were involved in “biosynthesis of other secondary metabolites,” “signal transduction,” and “environmental adaptation” pathways (Supplementary Figure S2).

### Construction of coexpression networks and identification of cultivation-induced hub genes

**Figure 5:**
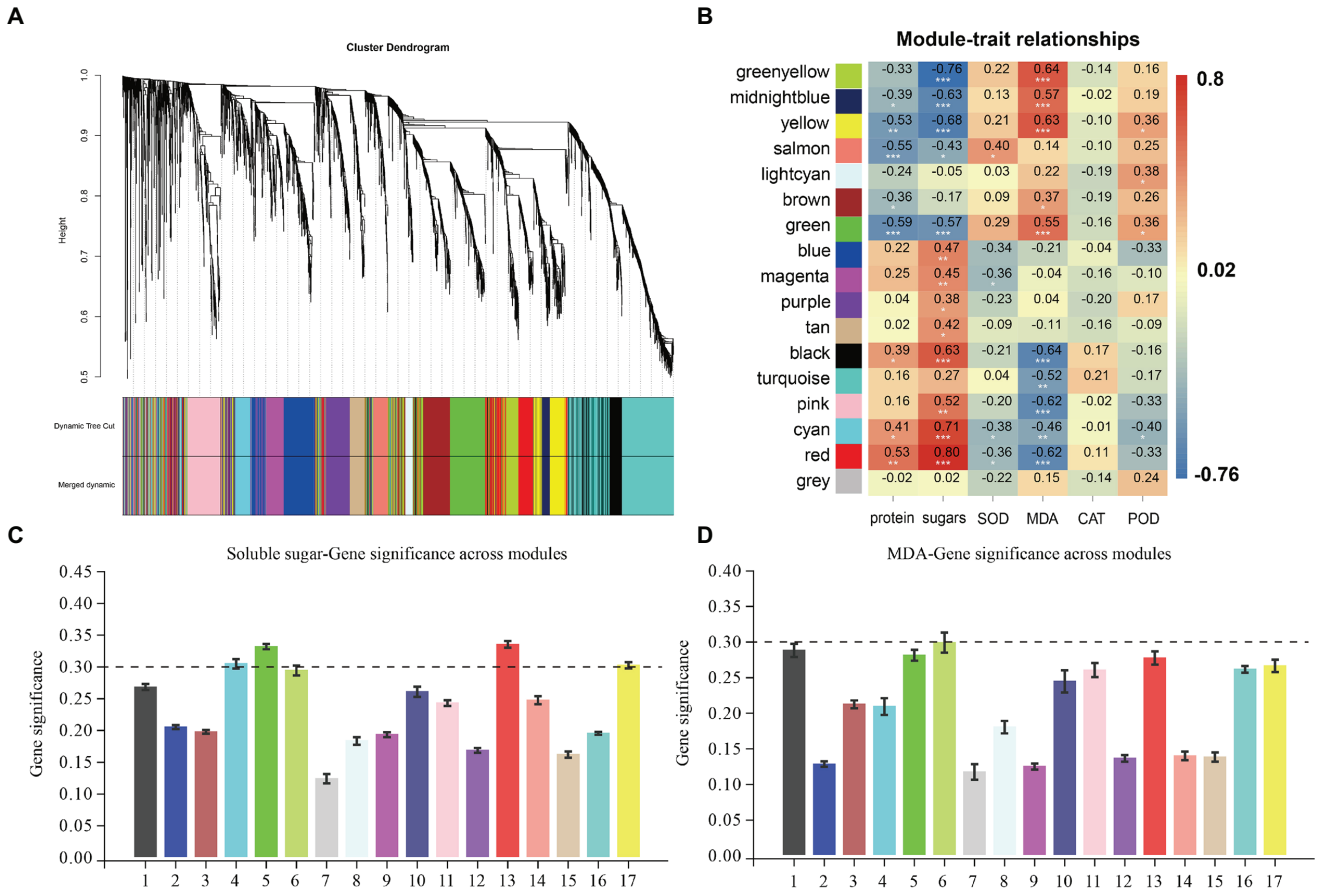
WGCNA coexpression network and module-trait correlation analyses. **(A)** The hierarchical cluster tree shows the coexpression modules identified by the dynamic tree cut method. Different modules are marked with different colors. Each leaf in the tree represents one gene. The main branches constitute 17 modules and are labeled with different colors. **(B)** Correlations between physiological indices and WGCNA modules. Each line corresponds to a module marked with the same color as in **(A)**. The column corresponds to the physiological index, and the color of each cell indicates the correlation coefficient between the module and the physiological index (the number in the cell represents the correlation coefficient, and the asterisk represents the *p* value). **(C)** Correlations between soluble sugar and WGCNA modules. **(D)** Correlations between MDA and WGCNA modules.

WGCNA was performed to further investigate the *Q. mongolica* regulatory network in response to the three cultivation measures and identify the specific genes that are strongly correlated with cultivation-induced physiological changes in *Q. mongolica*. Using the 3,672 DEGs generated by RNA-seq as source data, we constructed a scale-free coexpression network based on the soft-thresholding power of *β* = 6 (Supplementary Figure S3). According to the WGCNA results, clusters with highly interconnected genes were defined as modules, and genes in the same module were highly correlated. A total of 17 modules (Figure 5A) were identified *via* the dynamic tree cut method (merge cut height = 0.1). The number of genes in each module ranged from 27 to 692 (Supplementary Figure S4), and the gray module was a set of genes not assigned to any other module (27 genes). Correlation analyses showed that the green-yellow, midnight blue, yellow, green, black, pink, cyan, and red modules had significant positive or negative correlations with the physiological indices (Figure 5B). We obtained the average correlation value between genes in each module by calculating the correlation between the gene and the trait data and analyzed the correlation between each module and trait. With a genetic significance (GS) cutoff threshold ≥0.30 (Figures 5C,D), and in significantly related modules, the red (271 genes; *R* = 0.80, *p* < 0.05), cyan (104 genes; *R* = 0.71, *p* < 0.05), green (287 genes; *R* = −0.57, *p* < 0.05), and yellow (302 genes; *R* = −0.68, *p* < 0.05) modules were significantly correlated with soluble sugar. The green-yellow module (124 genes; *R* = 0.64, *p* < 0.05) was positively correlated with MDA.

**Figure 6:**
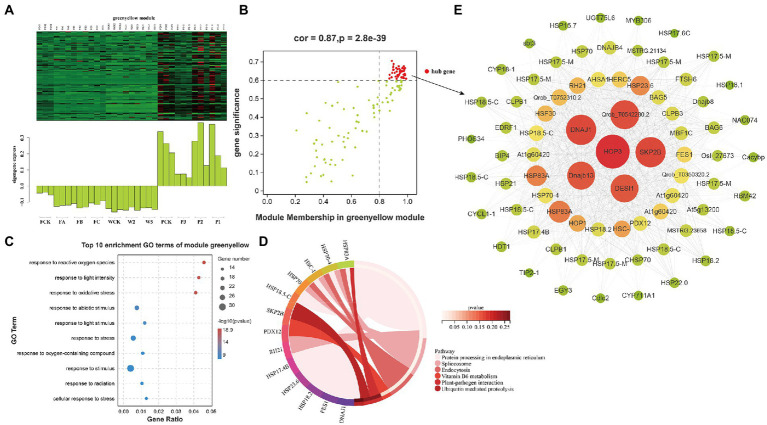
Coexpression network of the green-yellow module. **(A)** Gene coexpression heat map of the green-yellow module (upper panel) and expression of the corresponding eigengenes in each sample (lower panel). **(B)** MDA and the green-yellow module have a point map of the hub gene with higher connectivity and expression. The x axis represents the correlation between the expression of each gene and the module, and the y axis represents the correlation between each gene and the trait. **(C,D)** GO terms and KEGG enrichment analysis cycles of the top 10 hub genes associated with MDA traits in the green-yellow module. **(E)** Coexpression network of hub genes in the green-yellow module. The first 100 hub genes with a weight value >0.30 and higher expression were used to construct the network.

**Figure 7:**
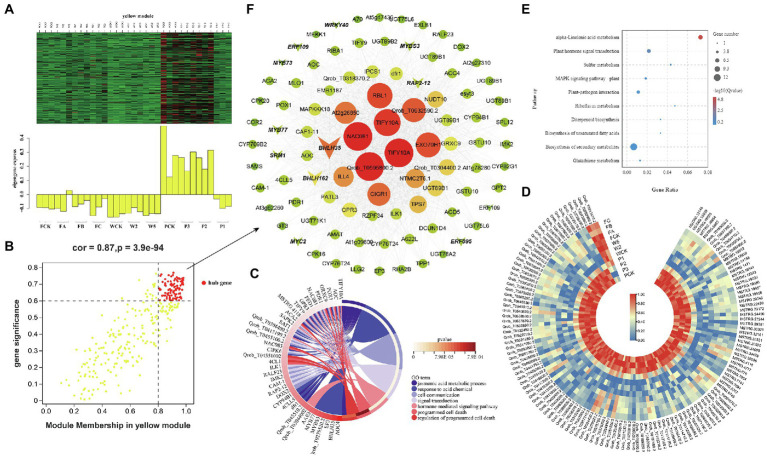
Coexpression network of the yellow module. **(A)** Gene coexpression heat map of the yellow module (upper panel) and expression of the corresponding eigengenes in each sample (lower panel). **(B)** Soluble sugar and the yellow module have a point map of the hub gene with higher connectivity and expression. The x axis represents the correlation between the expression of each gene and the module, and the y axis represents the correlation between each gene and the trait. **(C)** GO term enrichment analysis cycle of hub genes associated with soluble sugar traits in the module. **(D)** Heat map of hub gene expression associated with soluble sugar traits. **(E)** KEGG enrichment bubbles are associated with soluble sugars. **(F)** Coexpression network of hub genes in the yellow module. The first 100 hub genes with a weight value >0.30 and higher expression were used to construct the network.

**Figure 8:**
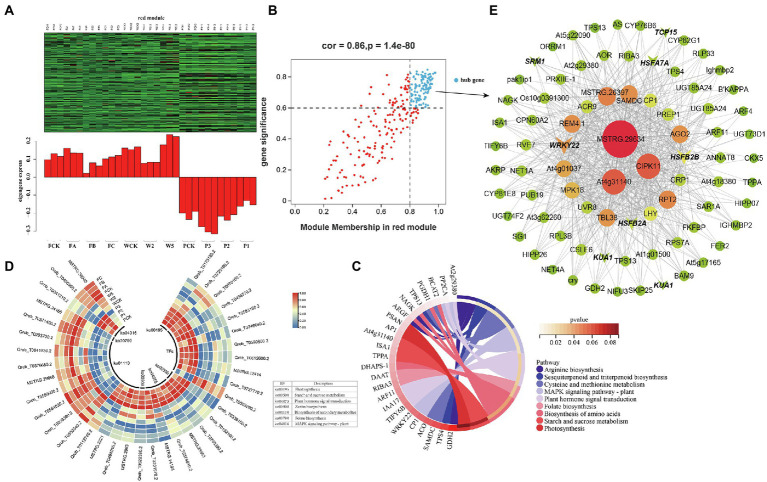
Coexpression network of the red module. **(A)** Gene coexpression heat map of the red module (upper panel) and expression of the corresponding eigengenes in each sample (lower panel). **(B)** Soluble sugars and the red module have a point map of the hub gene with higher connectivity and expression. The x axis represents the correlation between the expression of each gene and the module, and the y axis represents the correlation between each gene and the trait. **(C)** KEGG enrichment loop diagram of hub genes related to soluble sugar traits in the module. **(D)** Heat map of hub gene expression associated with soluble sugar traits. **(E)** Coexpression network of hub genes in the red module. The first 100 hub genes with a weight value >0.30 and higher expression were used to construct the network.

The first 20 KEGG pathways of the associated modules are shown in a bubble graph to provide more detail on the related modules (Supplementary Figure S5). Genes in the red and cyan modules were significantly enriched in the metabolic biosynthetic pathway. Examples include “starch and sucrose metabolism,” “photosynthesis,” “biosynthesis of amino acids,” and “sesquiterpenoid and triterpenoid biosynthesis.” Genes in the yellow and green modules were significantly enriched in “plant hormone signal transduction,” “glutathione metabolism,” “MAPK signaling pathway—plant,” and “plant–pathogen interactions.” In addition, after we summarized the expression patterns of characteristic genes in related modules, the genes in the green-yellow and yellow modules were positively correlated with P and negatively correlated with the amount of F and W (Figures 6A, 7A). The genes in red were positively correlated with F and W but negatively correlated with the P methods (Figure 8A). This result suggests that resistance genes in the two modules may regulate the stress response to different cultivation practices. The genes in which the modules were clustered were strongly correlated with their respective modules. In this study, hub genes were identified based on the MM > 0.8 and GS > 0.6 cutoff thresholds. After treatment with the different cultivation measures, the hub genes were expressed significantly differently in all samples. These genes were then analyzed for function. The hub genes obtained were called DEHGs. The green-yellow, red, and yellow modules were selected for coexpression network analyses to reveal the hub genes in the interaction between *Q. mongolica* and the different cultivation measures. Most genes only interact with a limited number of genes in a coexpression network; fewer genes (hub genes) interact with many other genes. There is no doubt that the more connected hub genes in a network play a central role. Thus, Cytoscape was used to construct the gene networks of the green-yellow, red, and yellow modules (weight > 0.3 and the first 100 genes) to understand the relationships among the genes in the modules.

### Functional enrichment analyses of hub genes correlated with the accumulation of MDA

Malondialdehyde is an important index of membrane lipid peroxidation. Functional analyses of hub genes identified 44 DEHGs in the green-yellow module as being closely related to MDA (Figure 6B; Supplementary Table S2-1). Consistent with these results, these hub genes were highly enriched in GO terms involved in the biological stress response, including “response to stress” (GO: 0006950), “response to ROS” (GO: 0000302), “response to light intensity” (GO: 0009642), “response to oxidative stress” (GO: 0006979), and “response to abiotic stimulus” (GO: 0009628; Figure 6C). The expression of DEHGs decreased under the W and F treatments but increased under the P treatments. KEGG pathway analyses (Figure 6D) showed that the DEHGs were enriched in metabolic and genetic information processes. The main enrichment pathways were “protein processing in endoplasmic reticulum” (12 DEHGs), “spliceosome” (4 DEHGs), and “endocytosis” (3 DEHGs). A gene coexpression network map was constructed, and the genes with high connectivity included HOP3, SKP2B, DNAJ1, DESI1, Dnajb13, HSP83A, HSP23.6, HSC-I, and HSF30 (Figure 6E).

### Functional enrichment analyses of hub genes correlated with the accumulation of soluble sugar

The red and cyan modules were positively correlated with soluble sugar under the different cultivation measures; 111 and 22 DEHGs (Figure 8B; Supplementary Tables S2-2,3) were identified, and nine TF genes were screened (Table 2). Eight genes encoding TFs (e.g., WRKY, MYB, HSF, Trihelix, and TCP) were positively expressed during P (Figure 8D). The main GO enrichments involved “nucleic acid binding transcription factor activity” (GO: 0001071), “response to stimulus” (GO: 0050896), and “response to ROS” (GO: 0000302). WRKY22 (MSTRG.12414) and HSF (Qrob_T0590800.2) had relatively high connectivity in the coexpression networks, which indicates that WRKY and HSF play a central role in the response to P measures (Figure 8E). In addition, five DEHGs were associated with the hormone category. According to KEGG pathway annotation of the hub genes, P induced expression of IAA17, TIFY6B, ARF11, and AT2G29380 (Figure 8D), which are related to “plant hormone signal transduction” (ko04075) and “MAPK signaling pathway—plant” (ko04016; Figure 8C). Many of these biosynthetic and metabolism processes changed in response to the different cultivation practices. According to the notes on DEHGs, most genes were involved in biosynthesis and metabolism, including 14 genes related to “biosynthesis of secondary metabolites,” three genes related to “starch and sucrose metabolism,” two genes related to “photosynthesis,” and one gene inducing “zeatin biosynthesis” and “folate biosynthesis” (Figure 8D). The coexpression network of the red module showed that the genes with high connectivity were MSTRG.29634, At4g31140, CIPK11, AGO2, RPT2, and MPK16 (Figure 8E).

**Table 2:** The identified transcription factors in the sugars-related modules.

Gene ID	Symbol	TF	Module	Description
MSTRG.12414	WRKY22	WRKY	Red	WRKY transcription factor 22 [*Quercus lobata*]
Qrob_T0070100.2	KUA1	MYB_related	Red	transcription factor MYB1R1 [*Q. lobata*]
Qrob_T0496770.2	TCP15	TCP	Red	transcription factor TCP14-like [*Q. lobata*]
Qrob_T0503290.2	SRM1	MYB_related	Red	transcription factor MYBS1 [*Q. lobata*]
Qrob_T0583150.2	HSFA7A	HSF	Red	Heat shock transcription factor [*Trema orientale*]
Qrob_T0590800.2	HSFB2B	HSF	Red	heat stress transcription factor B-2b [*Q. lobata*]
Qrob_T0612600.2	HSFB2A	HSF	Red	heat stress transcription factor B-2a [*Q. lobata*]
Qrob_T0727770.2	KUA1	MYB_related	Red	transcription factor MYB1R1-like [*Q. lobata*]
Qrob_T0746640.2	GT-2	Trihelix	Red	trihelix transcription factor GTL1 isoform X1 [*Q. lobata*]
MSTRG.12144	SRM1	MYB	Yellow	transcription factor SRM1 [*Q. lobata*]
MSTRG.15539	BHLH162	bHLH	Yellow	transcription factor bHLH162-like [*Q. lobata*]
MSTRG.18653	RAP2-12	ERF	Yellow	ethylene-responsive transcription factor ERF098-like [*Q. lobata*]
MSTRG.18654	ERF109	ERF	Yellow	ethylene-responsive transcription factor ERF098-like [*Quercus suber*]
MSTRG.5893	BHLH35	bHLH	Yellow	transcription factor bHLH35-like [*Q. lobata*]
Qrob_T0163650.2	ERF095	ERF	Yellow	ethylene-responsive transcription factor ERF098-like [*Q. lobata*]
Qrob_T0357390.2	MYBS3	MYB_related	Yellow	transcription factor MYBS3 [*Q. lobata*]
Qrob_T0468060.2	MYB77	MYB	Yellow	transcription factor MYB73-like [*Q. lobata*]
Qrob_T0687690.2	MYC2	bHLH	Yellow	transcription factor MYC2 [*Q. lobata*]

The green and yellow modules were negatively correlated with soluble sugar, and 36 and 94 DEHGs were identified in the modules, respectively (Figure 7B; Supplementary Tables S2-4,5). GO enrichment analyses showed that DEHGs played different roles in various biological processes, including “jasmonic acid metabolic processes” (GO: 0009694), “response to acid chemicals” (GO: 0001101), “cell communication” (GO: 0007154), “signaling” (GO: 0023052), and “response to stimulus” (GO: 0050896; Figure 7C). Most genes were induced in Zck, Z3, and Z2 treated with P and were inhibited in Z1, W and F treat, including nine genes encoding TFs (e.g., bHLH, ERF, MYB, and MYB; Figure 7D). KEGG enrichment analyses showed that the DEHGs were mainly concentrated in the “biosynthesis of secondary metabolites” and “plant hormone signal transduction” pathways (Figure 7E). Studies have shown that SRM1 (MYB) directly targets abscisic acid (ABA) biosynthesis and signal transduction–related genes (Wang et al., 2015), and ERF109 mediates crosstalk between JA signaling and auxin biosynthesis to regulate lateral root formation (Cai et al., 2014). Three genes associated with “protein processing in endoplasmic reticulum” and “plant–pathogen interaction” pathways, pfh1 (Qrob_T0043960.2), HSP70 (Qrob_T0252960.2), and CPK20 (Qrob_T0313470.2), were upregulated in the different W and F treatments. The genes with high connectivity in the yellow module were TIFY10A (MSTRG.38006), TIFY10A (Qrob_T0585450.2), NAC081 (Qrob_T0657980.2), EXO70H1 (Qrob_T0540670.2), and CIGR1 (Qrob_T0257560.2; Figure 7F). In summary, the annotation of gene function in the soluble sugar–related modules showed that stress-induced signal transduction occurred in *Q. mongolica* after the three cultivation treatments, which led to the accumulation of soluble sugars in the leaves.

### qRT-PCR validation of hub genes

**Figure 9:**
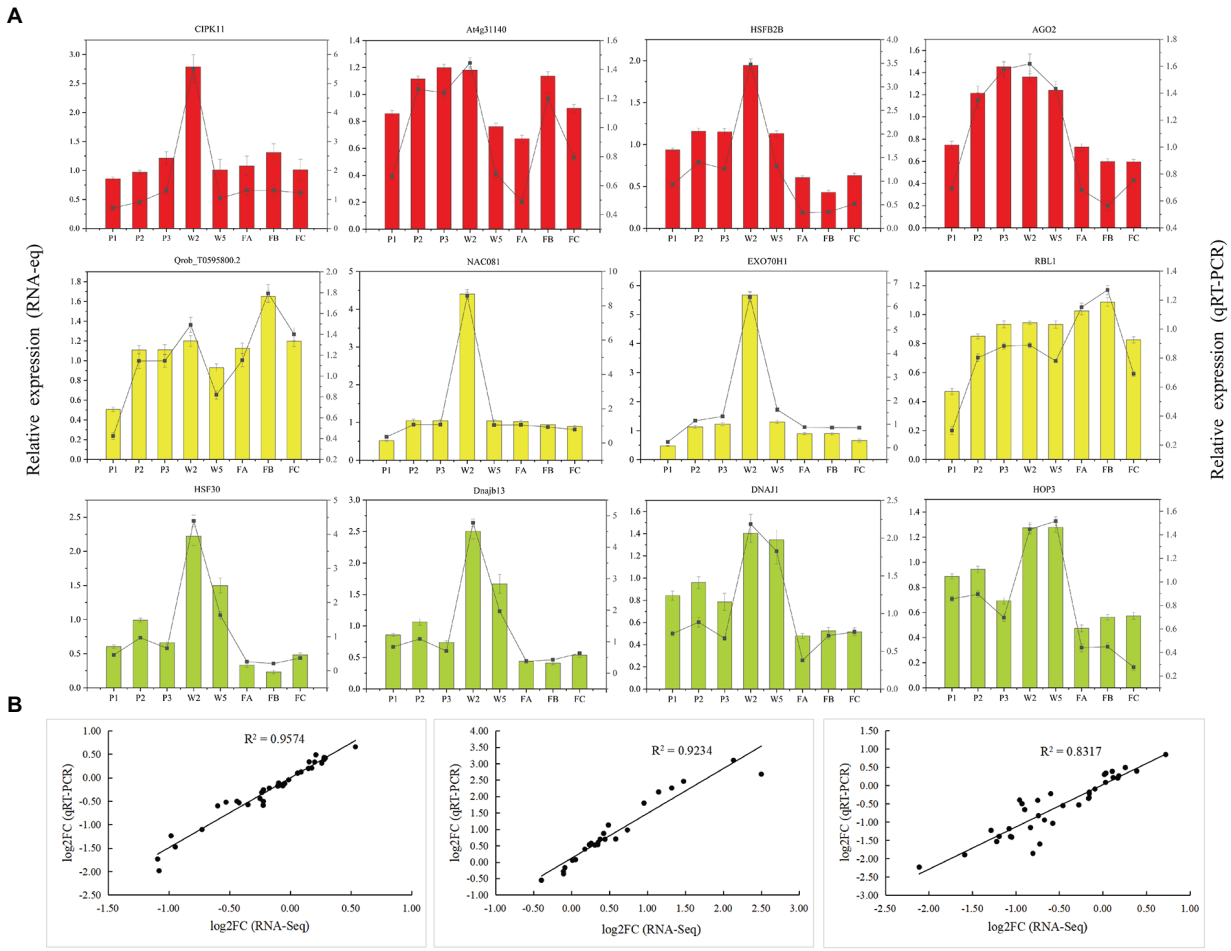
Validation of the selected genes in *Quercus mongolica* from RNA-Seq by qRT-PCR. **(A)** RNA-Seq and qRT-PCR results of hub genes from red, yellow and green-yellow modules. **(B)** Comparison of the log2 of gene expression ratios between RNA-Seq data and qRT-PCR results in P, W, and F treatments, respectively.

To validate the accuracy and reliability of the RNA-seq data, we performed qRT-PCR of 12 hub genes selected from three important WGCNA modules (red, green-yellow, and yellow). The 12 genes and their qRT-PCR primers are listed in Supplementary Table S3. The relative expression of the genes was highly consistent with the RNA-seq data (Figure 9A). Linear regression analyses revealed that the correlation between the RNA-seq and qRT-PCR data was significant (Figure 9B), which indicates that the RNA-seq data were accurate and credible.

## Discussion

### Growth response of *Quercus mongolica* under three cultivation measures

When *Q. mongolica* moved from high-altitude mountain area to plain area, the change of growth environment would affect the growth rhythm of trees, and demand for nutrients and water. Three treatments of fertilization amount 2–6 g/plant and five treatments of irrigation 1–5 times were set for *Q. mongolica*. To study the response of nutrient and water deficit, adequacy and surplus, and to determine the suitable fertilization amount and irrigation amount so as to provide reference for the quality of *Q. mongolica* seedlings. The height, diameter, and biomass of seedlings directly reflect the growth of a plant. Our results show that the growth of *Q. mongolica* seedlings responded significantly to the change of fertility level. The height, diameter and biomass of *Q. mongolica* seedlings increased significantly when the fertility level increased, but the promotion effect decreased when the fertilization level was too high, this is consistent with the results of Wang’s study (Wang et al., 2007). *Quercus mongolica* is not resistant to moisture, but suitable irrigation promotes radial growth (Lyu et al., 2017). Soil moisture increases photosynthesis during the growing season, which leads to the differentiation of more woody cells into wide rings (Fritts and Dean, 1992). The results show that W2 promoted the growth of *Q. mongolica* compared to the other treatments. *Quercus mongolica* has strong germination ability with the secondary growth characteristics and a great number of lateral branches, which leads to poor stem shape. It is the major problem in the development of *Q. mongolica* as a timber tree species (Wang et al., 2000). Pruning is the most direct and effective method, and the exploration of pruning technique can provide reference for the cultivation of good timber of *Q. mongolica* plantation in the future. Cutting significantly promoted the height of *Q. mongolica* seedlings because the plants grew faster and the biomass recovered rapidly after being damaged, a phenomenon called compensatory growth (Shi et al., 2000). Second, cutting off the original meristem increased the level of mitogens in the cells, which further stimulated the meristem and growth (Cipollini, 2002), which is consistent with the results of Yang for *Magnolia officinalis* (Yang et al., 2017).

### Physiological response of *Quercus mongolica* under three cultivation measures

Appropriate cultivation method can effectively promote the metabolism of physiological activities of trees, while unreasonable treatment would cause the growth retardation of trees. Under inappropriate cultivation measures, plant will undergo some physiological changes in order to maintain the stability of intracellular environment. Antioxidant enzymes and osmotic adjustment substances played important roles in maintaining the homeostasis of the plants under the different cultivation measures. But the basic mechanisms induced by the cultivation measures varied. As we all know, the activity of antioxidant enzymes is a symbol of ROS removal ability (Mutlu et al., 2013). SOD, POD, CAT, and other antioxidant systems are part of the upstream signaling pathway in plants. The SOD activity of the P-treated samples was higher than that of the W-or F-treated samples after cultivation. This finding indicates that all treatments induced the production of H_2_O_2_, and P activated the cellular antioxidant system and reduced harm. The results of OPLS-DA indicated that important *Q. mongolica* physiological regulatory factors differed under the different cultivation measures. SOD is the first line of defense against ROS. The SOD activity in the P treatment was in the order P1 > P2 > P3, which indicates that the P1 treatment promoted SOD activity. MDA is the main product of membrane lipid peroxidation, and its content directly reflects the degree of membrane peroxidation. In this experiment, MDA was important in the W treatment, as the increase in MDA content was higher in W5 than in FB and P2, which indicates that lipid peroxidation in W5 was more serious. This finding may be related to the observation that *Q. mongolica* is not resistant to humidity (Lyu et al., 2017; Zhao et al., 2021). Soluble sugars and soluble proteins are osmotic regulators in cells, and soluble sugars, including monosaccharides and oligosaccharides, are the main products of photosynthesis (Bodelón et al., 2010). Soluble sugars were the most important physiological substrate in the W treatment, which suggests that W adjusts the osmotic potential of cells by synthesizing soluble sugars to adapt to W. *Quercus mongolica* cannot tolerate humidity, so its photosynthetic activity is inhibited under water stress, which could explain why the accumulation of soluble sugars decreased with the increase in W. We have shown that the antioxidant system and osmotically important substances play a key role in the growth of *Q. mongolica* seedlings under different cultivation measures.

### Transcriptional differences in leaves under three cultivation measures

In this study, transcriptome changes in leaves of *Q. mongolica* were analyzed with RNA-Seq technique. 1,012, 1,175 and 1,035 DEGs were identified under P, W and F treatments, respectively. Gene expression varied under the different cultivation measures, and DEGs were most frequently detected in the P3, W2, and FC treatments. By comparing the GO enrichment analysis of the DEGs obtained from different cultivation treatments, we found that there were significant differences in biological functions among different cultivation treatments. This indicates that three different sets of genes were activated in the different cultivation measures. To establish the gene regulatory network and screen the central genes of *Q. mongolica* in different cultivation measures, we conducted WGCNA. This study aims to use the combined analysis of physiological and biochemical indexes and large-scale transcriptome data to recognize the gene expression modules of *Q. mongolica* in response to different cultivation measures (Figure 5B). WGCNA helped us get three important modules, among which the modules greenyellow and yellow specific genes were overexpressed in P; Modular red-specific genes were overexpressed in W and F. In addition, module greenyellow was significantly correlated with MDA, while module yellow and red were significantly correlated with soluble sugar (Figures 6A–8A). MDA was the final product of membrane lipid peroxidation (Campos et al., 2003; Cheng et al., 2020). We speculated that the upregulated genes in these modules would respond to the effects of different cultivation measures on the growth of *Q. mongolica* by affecting membrane lipid peroxidation and osmotic adjustment substances.

### Genes related to signal transduction under three cultivation measures

According to the plants demand, the experiment set different gradient cultivation methods to try to explore the best conditions for plant growth. If the plant cannot adapt to abiotic factors that exceed the threshold level, it would experience stress. Therefore, plants may trigger transcriptome reset in response to this environmental stimulus to ensure survival under given conditions. Plants rapidly transmit external signals to downstream components through a series of complex signal transduction pathways when faced with adverse environmental conditions, thus regulating their growth to better adapt to the changing environment. Ca^2+^ is another messenger for various signal transduction pathways in cells. Previous studies have identified proteins that are capable of sensing Ca^2+^ levels, including CaM, CDPK, and CBL. CBL interacts with CIPK to activate specific targets and transmit signals (Tang et al., 2015). CIPKs promote stress tolerance by modulating various physiological responses, and overexpression of OSCIPK12 induces the accumulation of proline and soluble sugars to improve the tolerance of rice to cold, drought, and salt stressors (Xiang et al., 2007). Overexpression of BrCIPK1 enhances proline biosynthesis in rice to increase tolerance to abiotic stress (Abdula et al., 2016). In this study, a CIPK11 gene with high connectivity was identified in the coexpression network of the red module, which indicates its potential role in the different cultivation practices. Protein kinase is of great importance in the signal pathway related to plant response to environmental stimuli. For instance, three protein kinases ATMEKK1, ATMPK3, and ATPK19 from *Arabidopsis thaliana* were activated by drought, injury and touch (Mizoguchi et al., 1996). In this study, At1g80180, an upregulated gene encoding MAPK, was found in W5, which indicated that MAPK signaling pathway could be activated under water stress. Plants also use hormonal balances of ABA, ethylene, and salicylic acid (SA) as signaling molecules in systemic acquired resistance. Jasmonic acid (JA) and other steroids also enhance tolerance and resistance to stress, and secondary metabolites, such as flavonoids, anthocyanins, lignin, and isoprene compounds, are produced and begin to accumulate (Wahid and Ghazanfar, 2006). The common CYP450 gene identified in the P experiments plays an important role in regulating homeostasis in secondary metabolism and hormone crosstalk between signaling molecules (Ahmad et al., 2019). In this study, 9 DEGs were in auxin, abscisic acid and ethylene signal transduction, and most plant hormones were upregulated in W5 and P3 treatments. It was indicated from the finding that the response of *Q. mongolica* to different cultivation methods involved various hormone signals.

### Genes related to metabolism under three cultivation measures

*Quercus mongolica* is often subjected to high temperature and strong light stress in summer when it is preserved and planted at low altitude. Studies have shown that heat stress induces the expression of HSPs, many of which can act as molecular chaperones to prevent protein degeneration and maintain protein balance in plants (Scharf et al., 2012). In addition, HSPs play a role in membrane stability by using ROS as a signaling molecule to actively regulate antioxidant enzymes (Chen et al., 2014; Driedonks et al., 2015). HOP3 (HSP70–HSP90), a member of the HOP family, interacts with its partner BIP and plays a major role in endoplasmic reticulum stress (Fernandez-Bautista et al., 2017). The dnaJ partner is also involved in the regulation of the heat shock response (Schroder et al., 1993). In this study, HOP3, dnaJ, and HSP genes with high connectivity in the green-yellow coexpression network were upregulated during the P treatment, which indicates that these genes have a potential role inpruning response. Carbohydrates, the main source of carbon and energy for cell building and consumption, are regulated to control metabolism, resistance to stress, and regulation of growth and development during the early stages of evolution (Sun et al., 2015). Sucrose is involved in the stress response, as it increases levels of metabolites and enhances the activity of specific enzymes (Klotz and Haagenson, 2008). Most of the metabolic pathways identified in this study were related to carbohydrate metabolism (starch and sucrose metabolism, amino acid and nucleotide metabolism, and galactose metabolism). The greatest number of DEGs were involved in FC and W2 processes. Sugars played an important role in F and W by maintaining membrane permeability, increasing cell fluid concentration, and supporting cell integrity. Protein levels associated with carbohydrate and energy production increase significantly in drought-tolerant and drought-sensitive cultivars of adzuki bean (Han et al., 2021), which suggests that carbohydrates are involved in increases in transcript and protein levels during the drought response.

### Major TFs involved in the response of three cultivation measures

As gene regulators, TFs play a key role in regulating gene expression and signal transmission in plant cells. In this study, 28 (5%) DEGs were annotated as TF, belonging to 9 different families (Supplementary Table S4), among 573 DEGs significantly related to soluble sugar. A large number of TFs have been identified as belonging to ERF, bHLH, MYB, WRKY, HSF families. A great number of TFs were regulated in all cultivation practices, while some TFs were specifically regulated in one treatment (Supplementary Figure S8). These TF families were considered to attach great importance in adapting to changing environmental conditions. For instance, it had potential functions in regulating drought, cold and salt stress response and improving drought, cold and salt tolerance of plants (Peng et al., 2015; Zhang et al., 2019, 2020a). Different TFs such as ERF, MYB, WRKY and HSF were also summarized, which were helpful to adapt to various abiotic stresses by sensing signals, switching stress response genes and regulating different signal transduction pathways (Zhou et al., 2009; Zhang et al., 2012; Alves et al., 2014). In this study, some members of the bHLH, MYB, WRKY, and HSF transcription factor families, such as bHLH35, WRKY22, HSFB2B, and SRM1, may also be involved in regulating the expression of genes in response to the cultivation measures. Most of these transcription factors were induced in the P experiments, and WRKY and HSF had higher connectivity in the coexpression network. WRKY transcription factors are involved in MAPK signaling and stress-induced defense responses (Jiang et al., 2017). The four differentially expressed WRKY transcription factors detected in this study which were highly induced in P3. It is indicated that the WRKY transcription factors recognized might be positively correlated with pruning-induced responses.

## Conclusion

The results of this study showed that cutting could effectively promote the longitudinal growth of *Q. mongolica*, irrigate two times during the growth period could promote the radial growth. And 4 g/plant (FB) was the optimal fertilization amount. However, the physiological and molecular mechanisms behind the response of *Q. mongolica* to three cultivation measures are complex. The physiological measurements in this study showed that P was more effective than W or F for activating intracellular antioxidant systems. By contrast, W and F were more effective than P for inducing the accumulation of soluble sugar. The essential physiology-regulating substances of the different cultivation measures were SOD and POD in P, MDA in W, and POD in F. The responses of *Q. mongolica* to the different cultivation measures were mediated by signal transduction, hormones, metabolism, functional proteins, and transcription factors. The proteins HOP3 and dnaJ, the heat shock protein genes, the signal transduction–related gene CIPK11, and the transcription factors WRKY, HSF, and bHLH were at the core of the network and may played a key role in the response of three cultivation measures. This study provides insight into the adaptation mechanism of *Q. mongolica* and other plants under cultivation.

## Data availability statement

The datasets presented in this study can be found in online repositories. The names of the repository/repositories and accession number(s) can be found at: https://www.ncbi.nlm.nih.gov/ PRJNA839552.

## Author contributions

MJ and XL are equal contributing authors for this manuscript. MJ, XL, JW, and MY designed the experiment. MJ, XL, YY, GZ, JP, and JR performed data and tissue collection. JW and MY conceived the study and edited the manuscript. All authors contributed to the article and approved the submitted version.

## Funding

This research was supported by the National Key R&D Program of China during the 14th Five-Year Plan Period (2021YFD2200302).

## Conflict of interest

The authors declare that the research was conducted in the absence of any commercial or financial relationships that could be construed as a potential conflict of interest.

## Publisher’s note

All claims expressed in this article are solely those of the authors and do not necessarily represent those of their affiliated organizations, or those of the publisher, the editors and the reviewers. Any product that may be evaluated in this article, or claim that may be made by its manufacturer, is not guaranteed or endorsed by the publisher.

## Footnotes

^1^ http://www.omicsmart.com/

^2^ https://www.omicshare.com/tools/Home/Soft/getsoft
