# Supplementary figures and images for: Integrated physiological and transcriptomic analyses reveal the molecular mechanism behind the response to cultivation in Quercus mongolica
Source: Front Plant Sci. 2022 Aug 8;13:947696. doi: 10.3389/fpls.2022.947696 (PMC9393570; doi:10.3389/fpls.2022.947696)

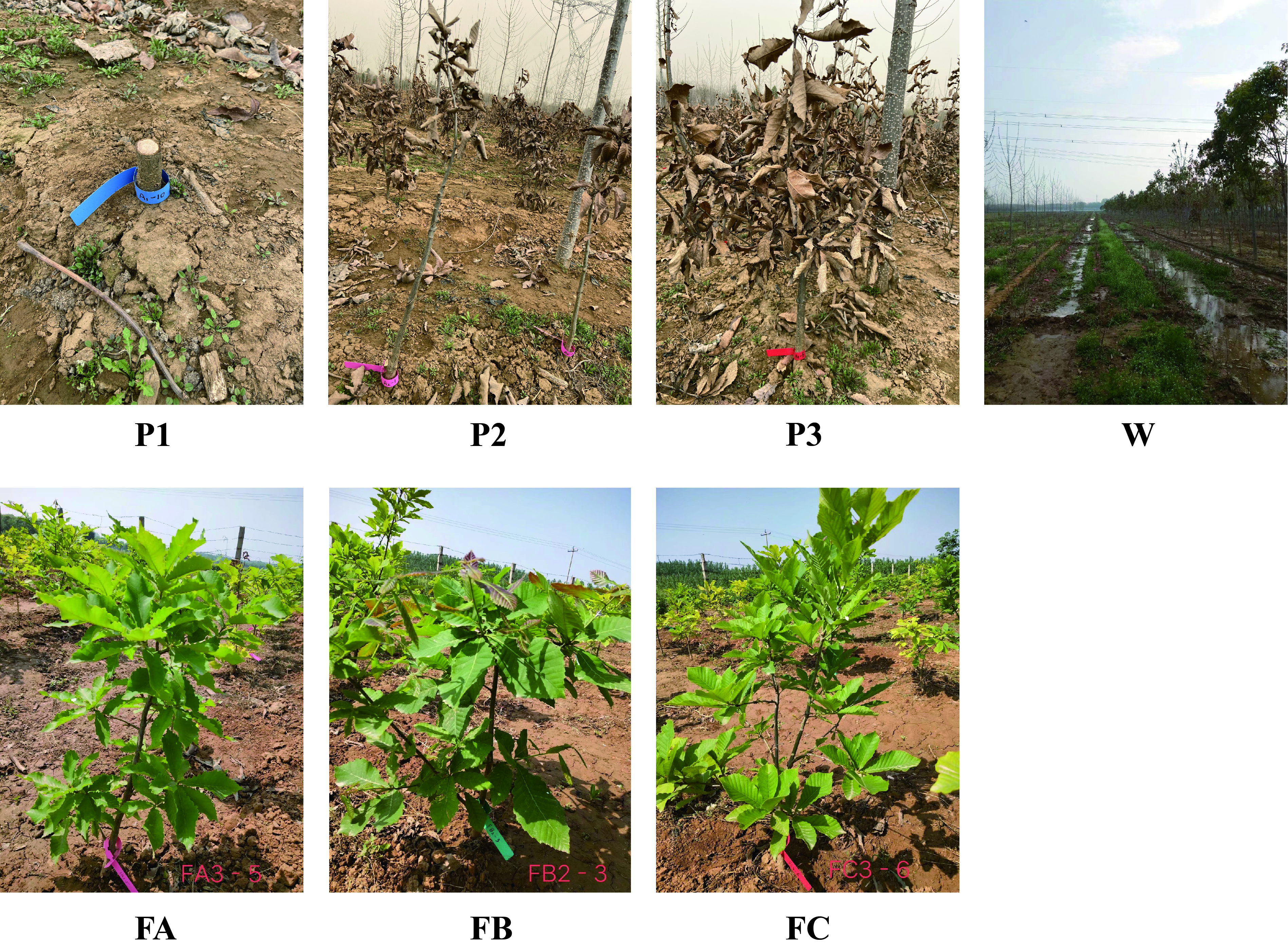

Supplement: Supplementary file 1 [file Image_1.JPEG]

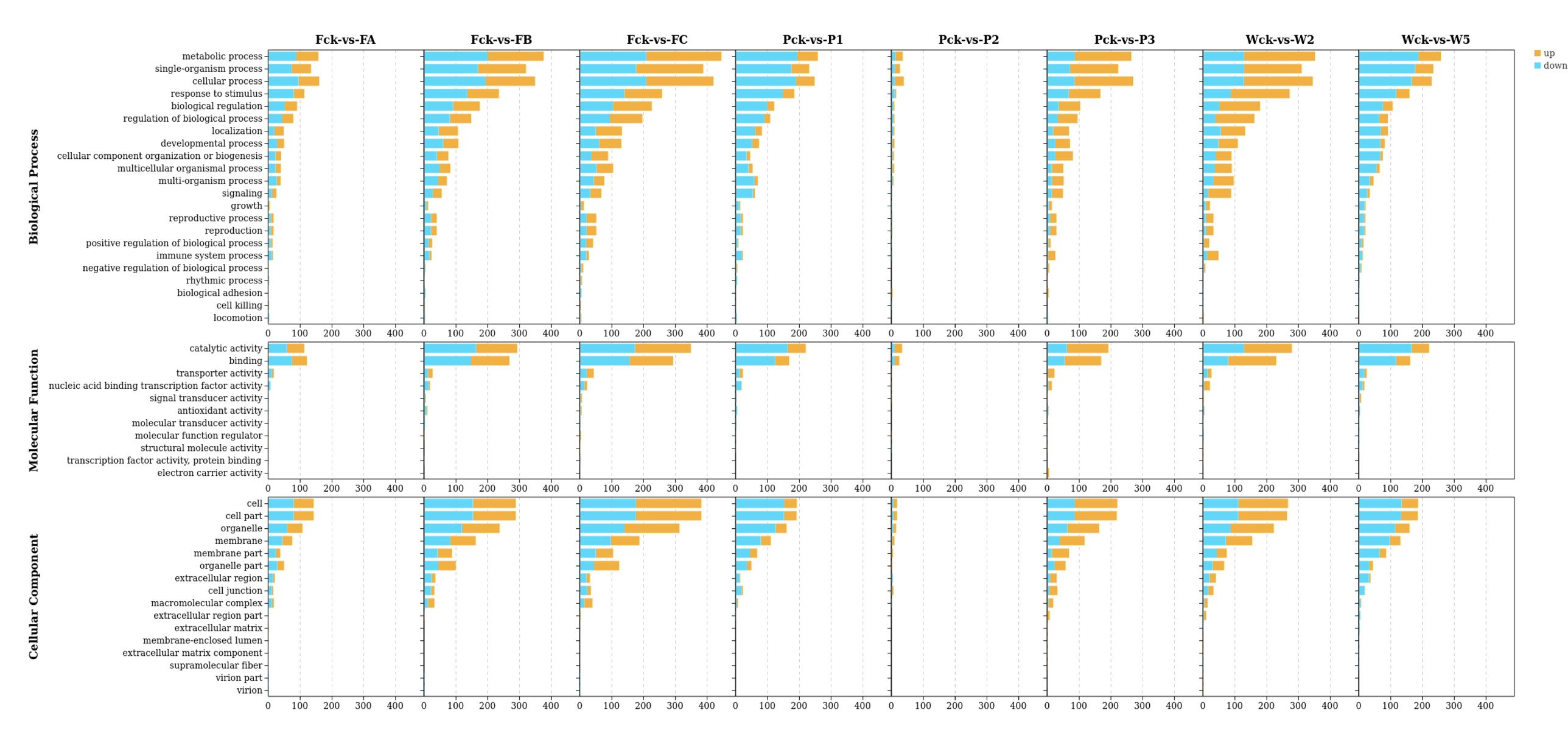

Supplement: Supplementary file 2 [file Image_2.JPEG]

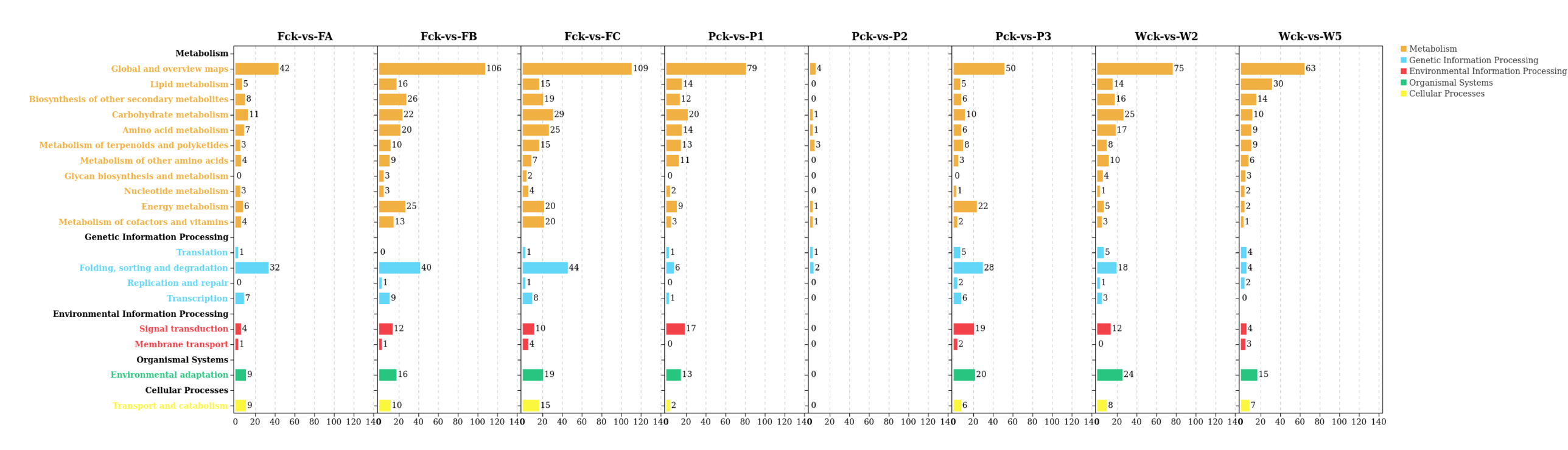

Supplement: Supplementary file 3 [file Image_3.JPEG]

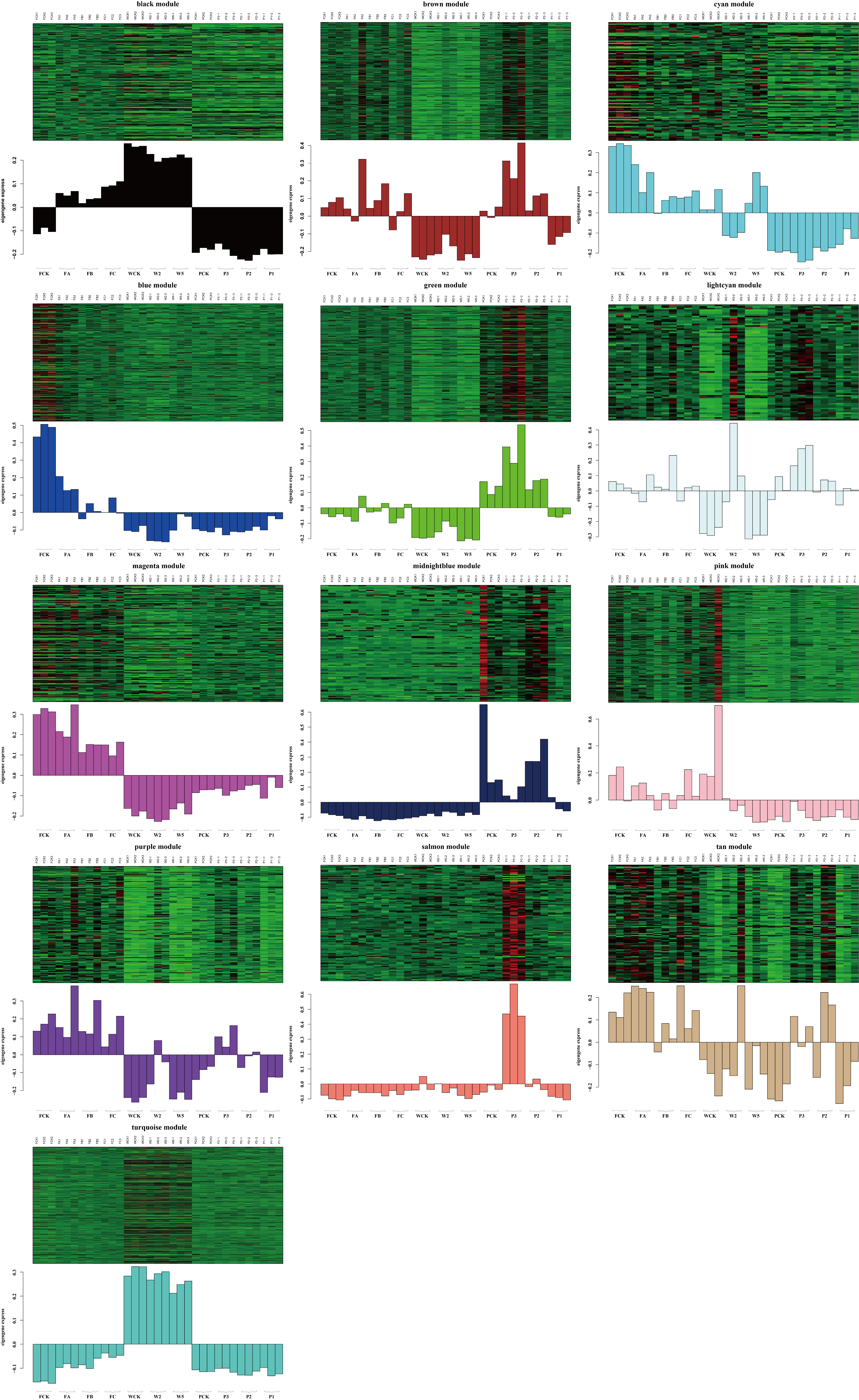

Supplement: Supplementary file 10 [file Image_10.JPEG]

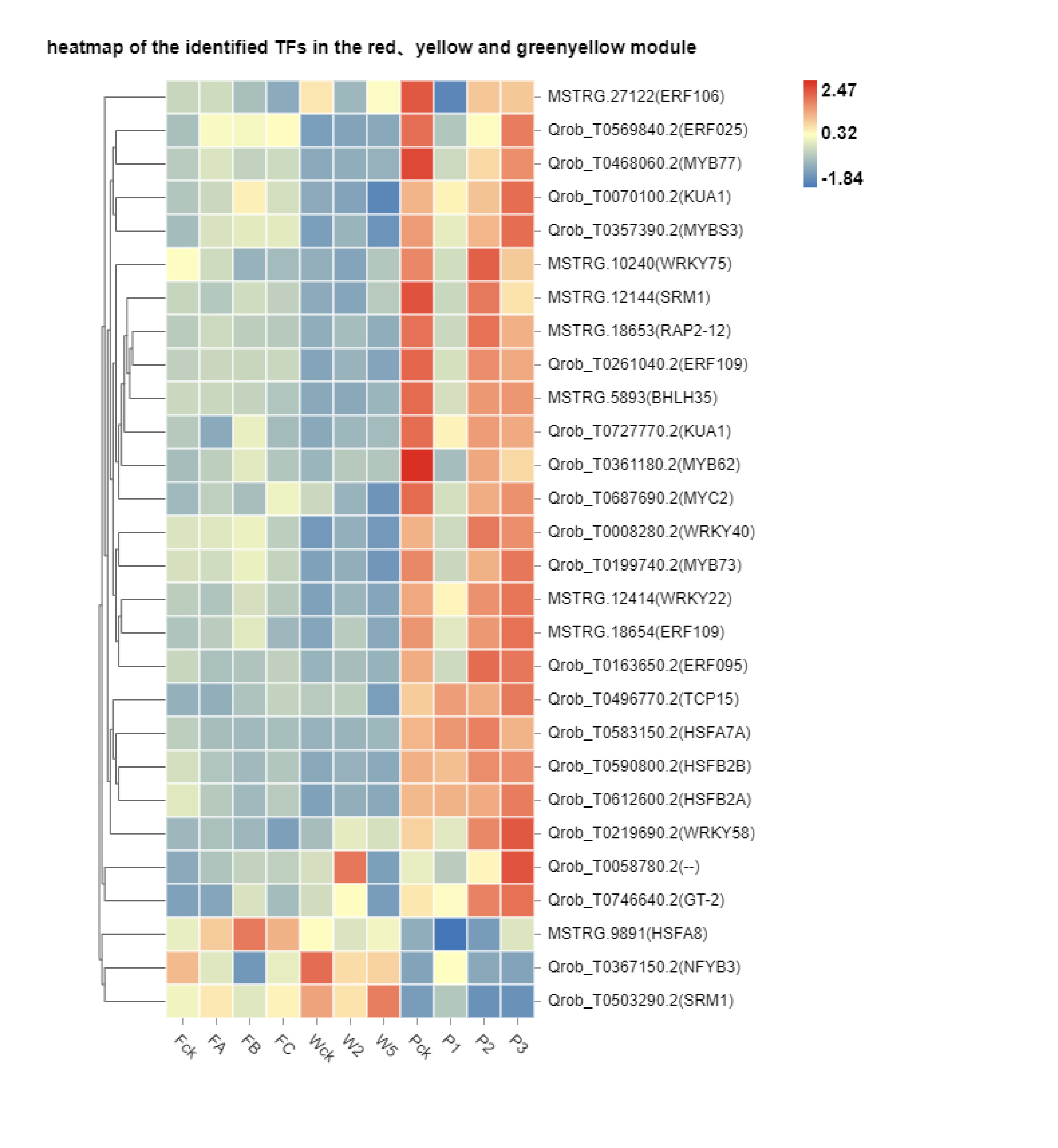

Supplement: Supplementary file 11 [file Image_11.JPEG]
